# Supplementary material for: Enhanced mitochondrial G-quadruplex formation impedes replication fork progression leading to mtDNA loss in human cells
Source: Nucleic Acids Res. 2023 Jun 23;51(14):7392–408. doi: 10.1093/nar/gkad535 (PMC10415151; doi:10.1093/nar/gkad535)
Supplement: gkad535_Supplemental_File [file gkad535_supplemental_file.pdf]

Sup.Figure 1

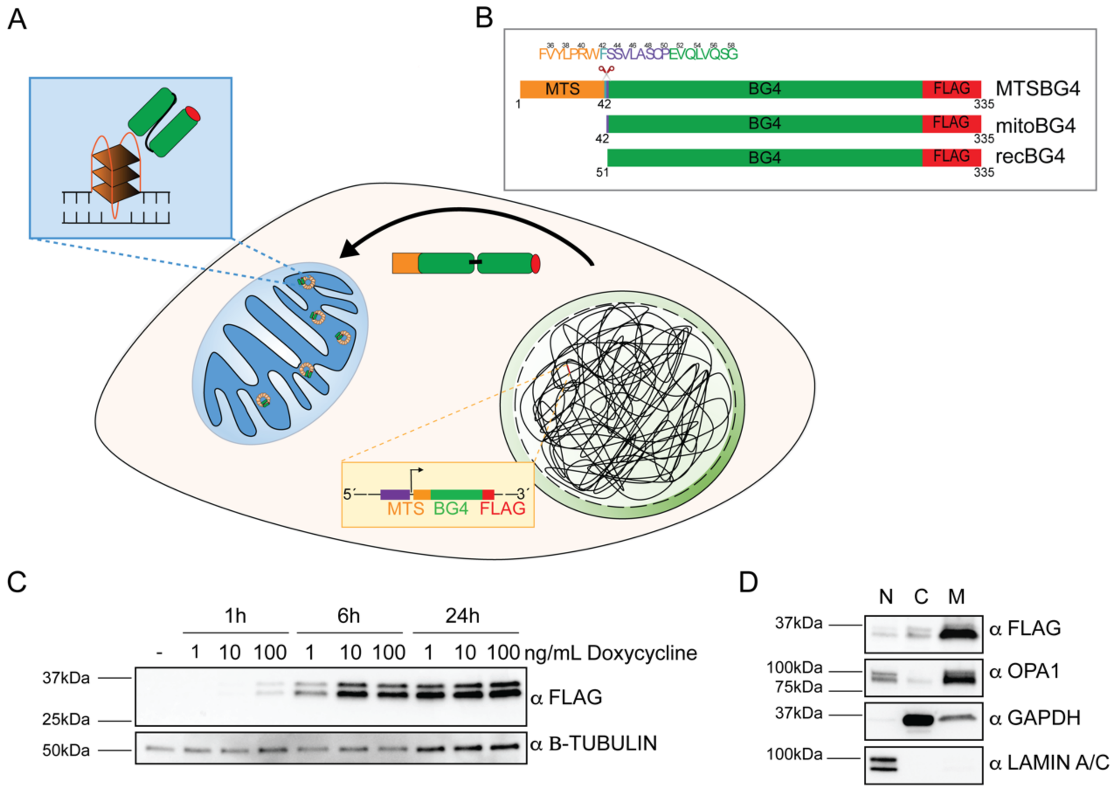

Sup.Figure 1: A. Schematic representation of the cell model employed in the study. Briefly, MTS-BG4-FLAG gene is integrated in the genome of the HEK Flp-In T-REx system under the control of an inducible promoter. Upon doxycycline treatment the protein is expressed and targeted to the mitochondrial matrix where, upon cleavage of the mitochondrial targeting sequence and proper folding, it can bind to G4 structures in the mtDNA. B. Schematic representation of MTS-BG4 processing into mitoBG4 and comparison with recombinant BG4 (recBG4). The site of processing is highlighted. C. Immunoblot of total cell extracts from mitoBG4 cells treated with different concentrations of doxycycline for the indicated period of time. Samples were probed with FLAG antibody to detect mitoBG4. B-TUBULIN antibody was used as loading control. D. Immunoblot of nuclear (N), cytosolic (C) and mitochondrial (M) fractions from induced mitoBG4 cells. Antibodies against LAMIN A/C (nucleus), GAPDH (cytosol) and OPA1 (mitochondria) were used to detect the purity of the different fractions.

Sup.Figure 2

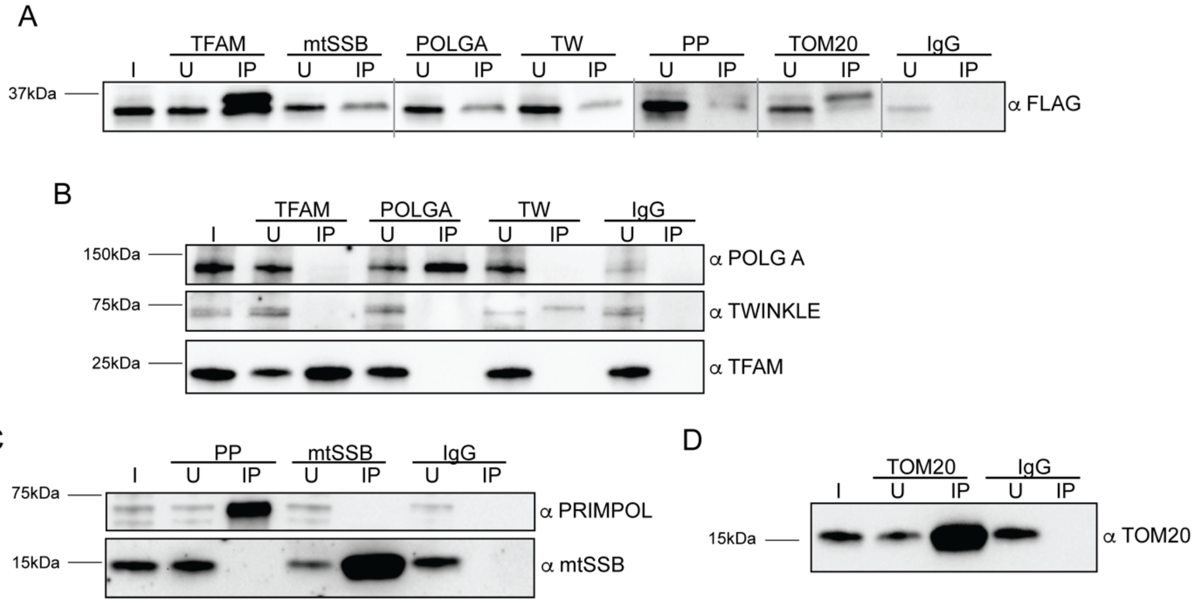

Sup.Figure 2: A. Immunoblot of INPUT (I), unbound (U) and pull-down (IP) fractions from immunoprecipitation with the indicated antibodies of induced mitoBG4 cells. The blot was probed with the FLAG antibody to detect mitoBG4. B, C and D. Immunoblot of INPUT (I), unbound (U) and pull-down (IP) fractions from immunoprecipitation with the indicated antibodies of induced mitoBG4 cells. PP: PRIMPOL, TW: TWINKLE.

Sup. Figure 3

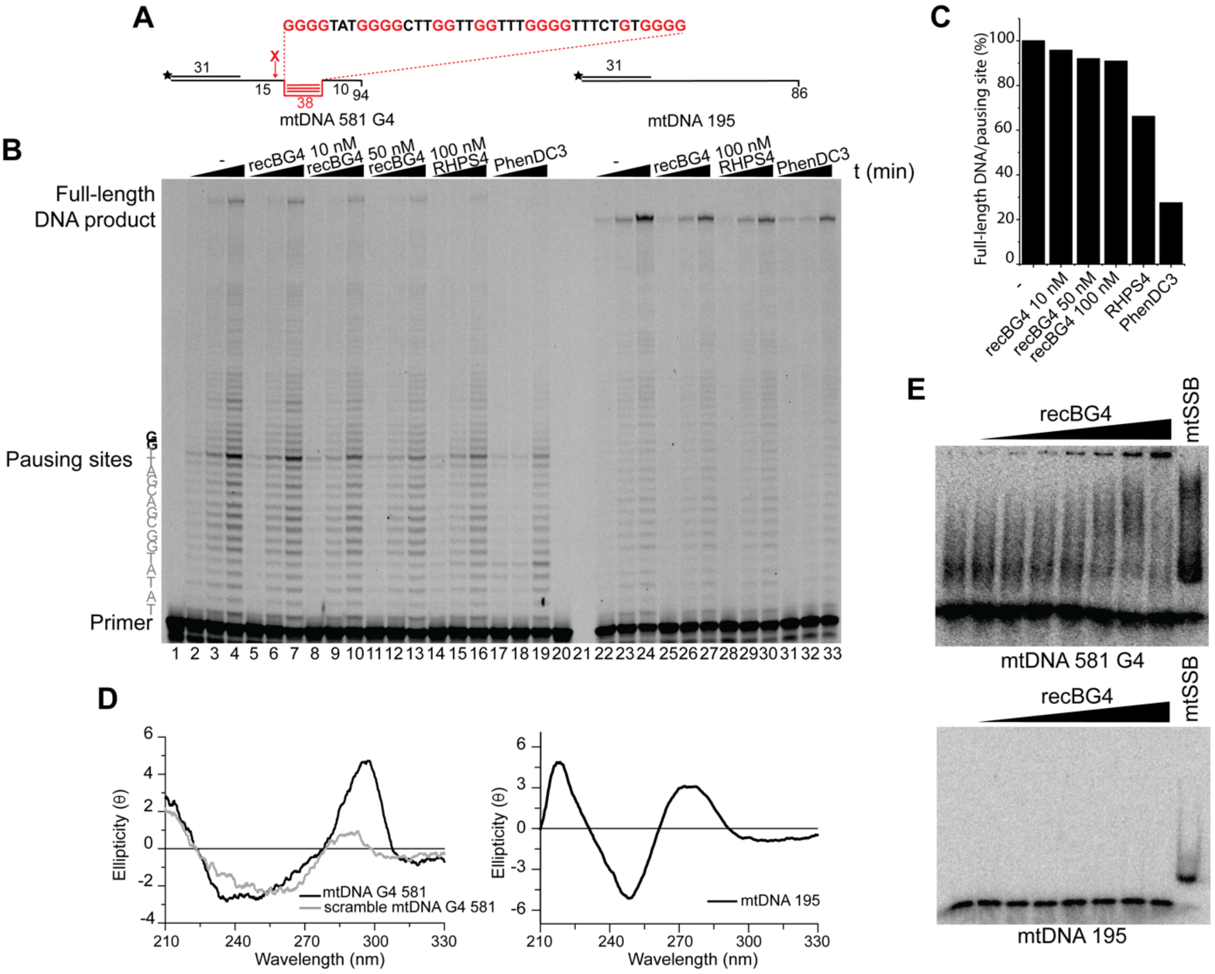

Sup.Figure 3: A. Schematic representation of the templates used for the polymerase stop assay. B. Polymerase stop assay on a template with a G4 structures in the presence of increasing concentration of recBG4. For each concentration, the reaction was blocked at increasing time points (1, 5 and 20 minutes). The template sequence before and around the pausing site are highlighted on the right side. Guanines involved in the G4 formation are indicated in bold. C. Relative ratio of the intensity of the full-length DNA product to G4 pausing site signal is plotted to show reduction in full length DNA product, confirming G4 stabilization. D. CD spectra of mtDNA G4-forming sequences 581 and mtDNA 195 (non G4 forming sequence) that were used in the Polymerase stop assay. A scrambled sequence with identical guanine content and length were used as control for mtDNA G4 581. 3 μM of the DNA template was folded in 100 mM KCl prior to spectra recording. The analyzed G4 displayed a profile indicative of a G4 structure with anti-parallel topology. Scrambled sequence (grey in the left graph) and mtDNA

195, present with a spectrum typical of dsDNA. E. Electrophoretic mobility shift assay (EMSA) was performed with 1 nM DNA substrates used in the Polymerase stop assay after incubation with increasing concentrations of recBG4. Recombinant mtSSB (mtSSB) was added as binding control.

Sup.Figure 4

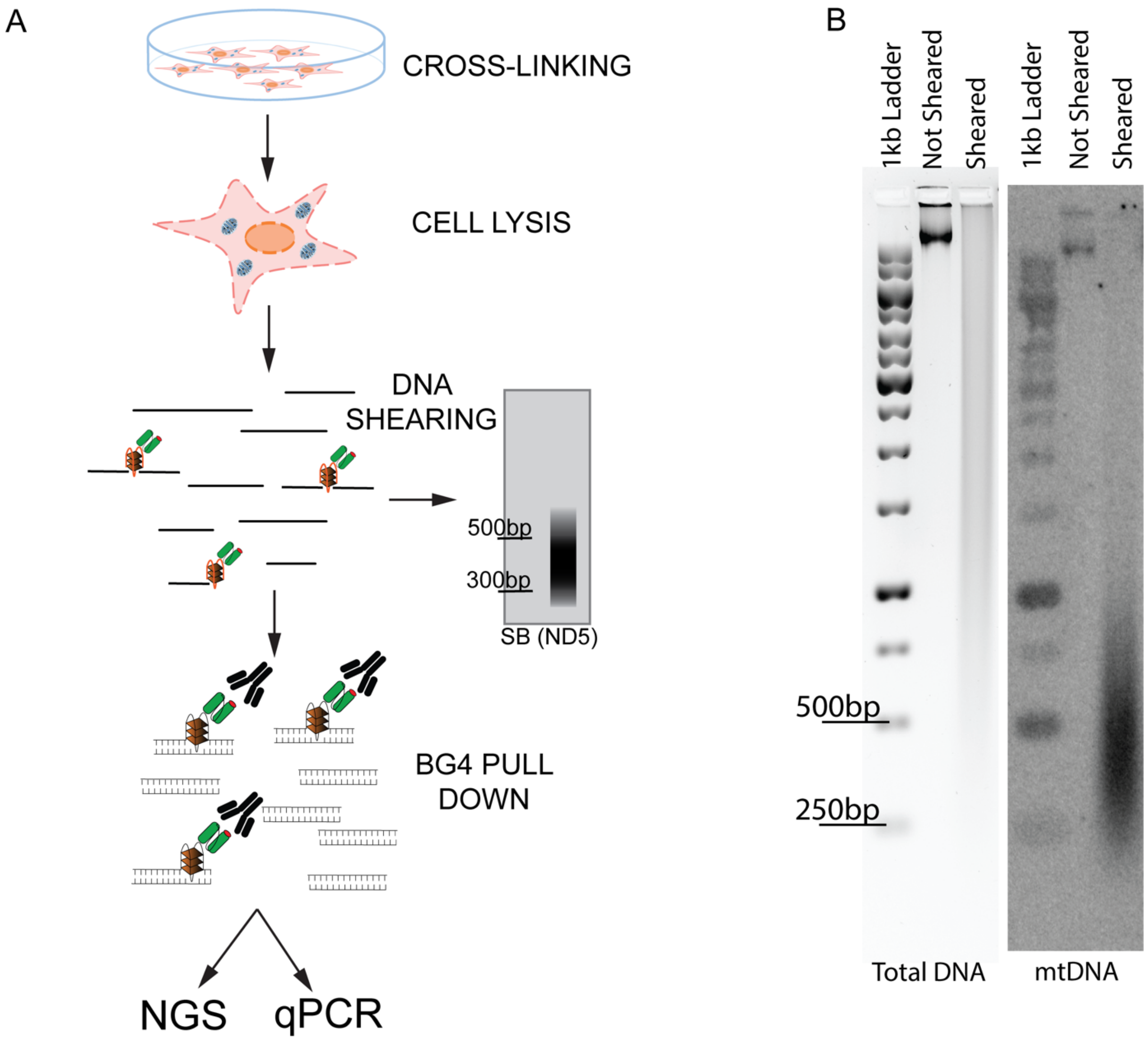

Sup.Figure 4: A. Schematic representation of the mtG4-ChIP protocol. B. Southern blot analysis of DNA shearing for the mtG4-ChIP. Reverse-cross linked DNA intact and sheared was separated on agarose gel and detected under UV light upon Ethidium bromide staining to detect total DNA signal (left picture-total DNA). The separated DNA was then blotted on nylon membrane and the mtDNA was detected with a ^{32}P-labelled mtDNA specific probe (against ND5 region, right picture-mtDNA).

Sup.Figure 5

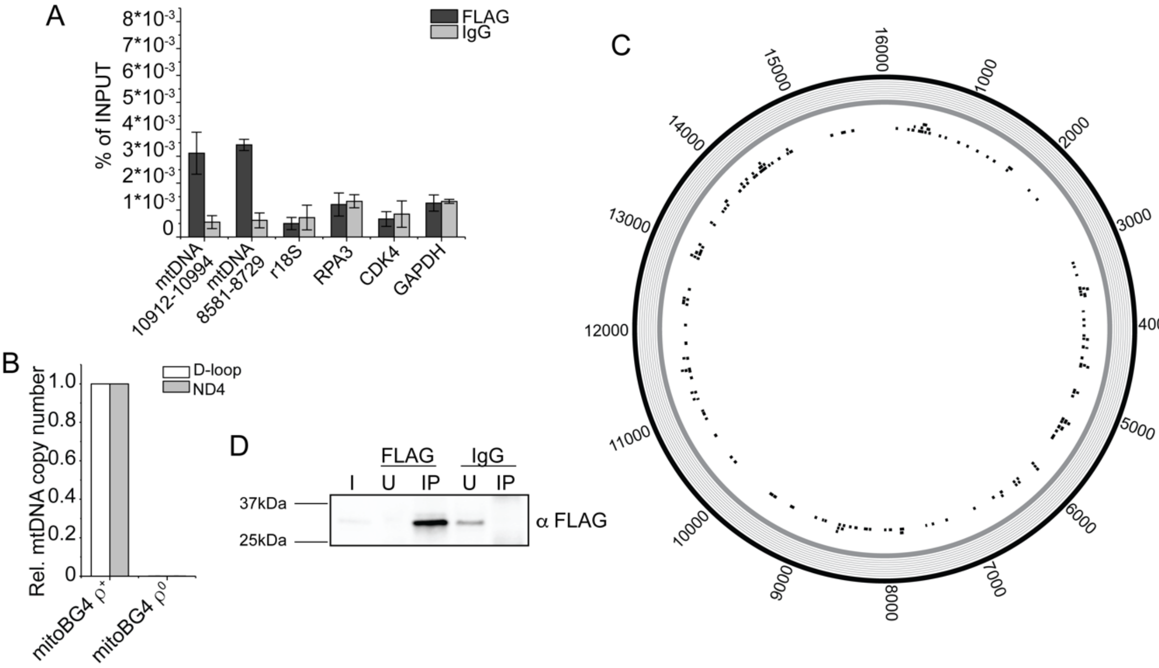

Sup.Figure 5: A. ChIP-qPCR upon pull down with FLAG and IgG antibodies in induced mitoBG4 cells. Two regions in the mtDNA (mtDNA 10912-10994 and mtDNA 8581-8729) not encompassing a predicted G4 sequence and four nDNA regions were analyzed. RPA3, CDK4 and GAPDH amplicons contain a predicted G4 sequence. Data are expressed as % of INPUT. Data represent mean ± s.d. of three independent experiments. B. mtDNA copy number of mitoBG4 ρ^+ and ρ^0 cells. MtDNA copy number was determined by qPCR from total DNA. Two different regions of the mtDNA, D-loop and ND4, were analyzed. The relative mtDNA copy number was expressed as the ratio between mtDNA regions and nuclear DNA B2M region and normalized to ρ^+ samples. Data represent mean ± abs error of two independent experiments. C. mtG4-ChIP-seq profile for induced mitoBG4 ρ^0 cells (lacking the mtDNA) pulled down with FLAG antibody. Grey color indicates no enrichment. The black boxes in the inner circle are predicted G4 sequences (using the G4 hunter algorithm). D. Immunoblot of Input (I), unbound (U) and pull-down (IP) fractions from FLAG and IgG immunoprecipitation of induced mitoBG4 ρ^0 cells. The blot was probed with the FLAG antibody. FLAG pull down fraction is enriched for mitoBG4.

Sup.Figure 6

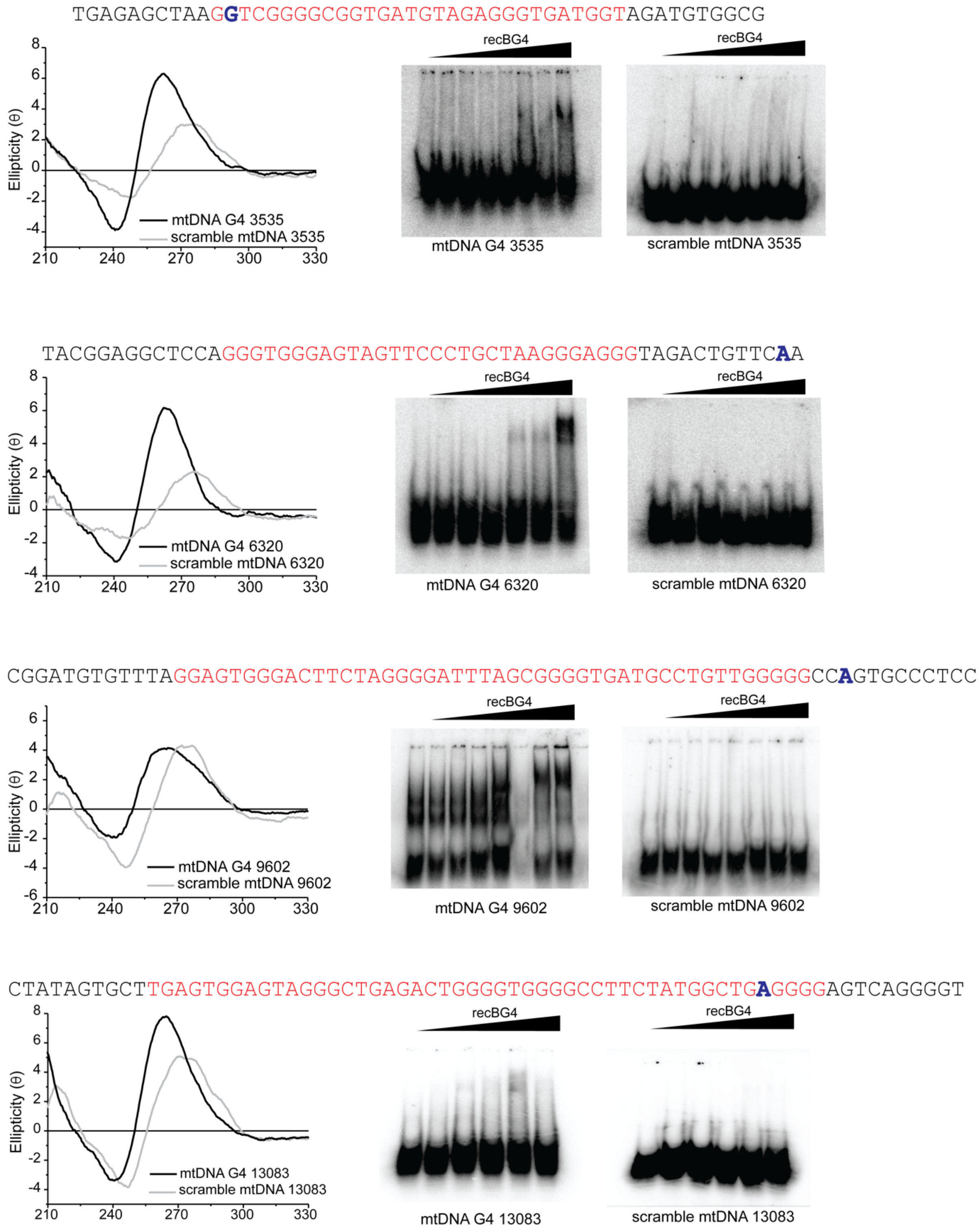

Sup.Figure 6: Left: CD spectra of putative G4-forming sequences identified in the mtG4-ChIP-seq protocol. 3 μM of template was folded in 100 mM KCl prior to spectra recording. Scrambled sequences with identical guanine content and length were used as control. All PGS analyzed displayed a positive peak at around 264 nm indicative of a G4 structure with parallel

topology. Scrambled sequences (grey), present with a spectrum typical of dsDNA. Right: EMSA was performed in the presence of increasing concentrations of recombinant BG4 (BG4) of the oligos used for the CD analysis. DNA concentration was 1 nM. Analyzed sequences are in red, bold letters indicate the peak summit as detected in mtG4-ChIP-seq analysis (see Sup. Table 1).

Sup.Figure 7

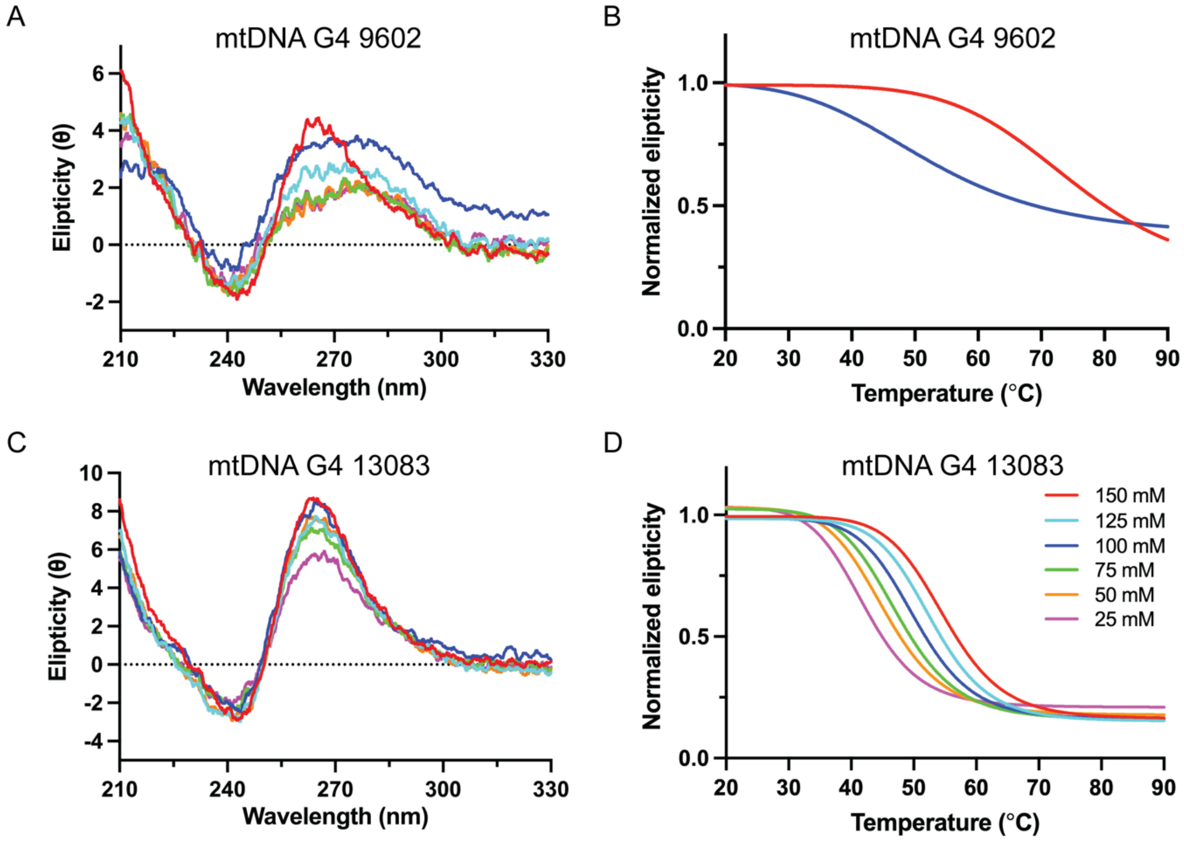

Sup.Figure 7: Circular dichroism (CD) (A and C) and thermal melting analyses of mtDNA (B and D) G4 9602 (A and B) and mtDNA G4 13083 (C and D). The colors indicate the different KCl concentrations used in the assays. DNA concentration was 1 μM.

Sup.Figure 8

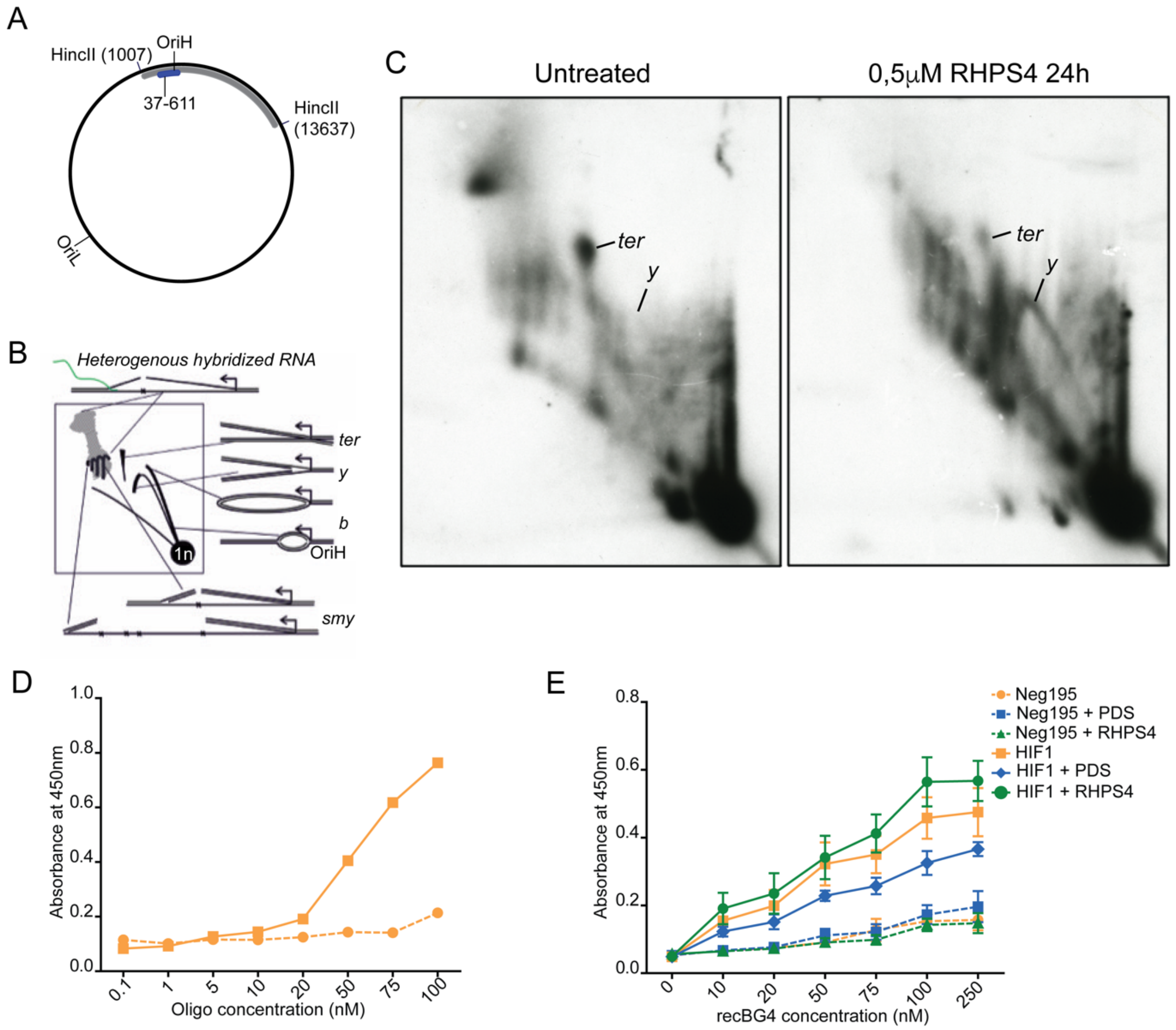

Sup.Figure 8: A. Schematic illustration of human mtDNA showing the HincII restriction sites used for linearization of mtDNA. The probe used for southern blot hybridization is indicated in blue. B. Graphical representation of the mtDNA replication intermediates that can be detected by 2D-AGE. C. 2D-AGE of HincII-digested mtDNA from HEK cells untreated (left) or treated with RHPS4 0,5 μM for 24 h, probed for the OriH-containing fragment as illustrated in A. A strong pausing site is visible upon RHPS4 treatment. D. recBG4 binding curves determined by ELISA for HIF1 (G4 forming) and neg195 (non G4 forming) oligos. The following oligos concentrations were used: 0.1, 1, 5, 10,20, 50, 75 and 100 nM. recBG4 concentration was 250nM E. recBG4 binding curves determined by ELISA for HIF1 (G4 forming, orange square) and neg195 (non G4 forming, orange circle) oligos in presence of the G4 ligands Pyridostatin (PDS) and RHPS4. Non G4 forming sequence (neg195) was used as negative control. The

following recBG4 concentrations were used: 0, 10, 20, 50, 75 and 100 nM. Data represent mean ± SEM of four independent experiments.

Sup.Figure 9

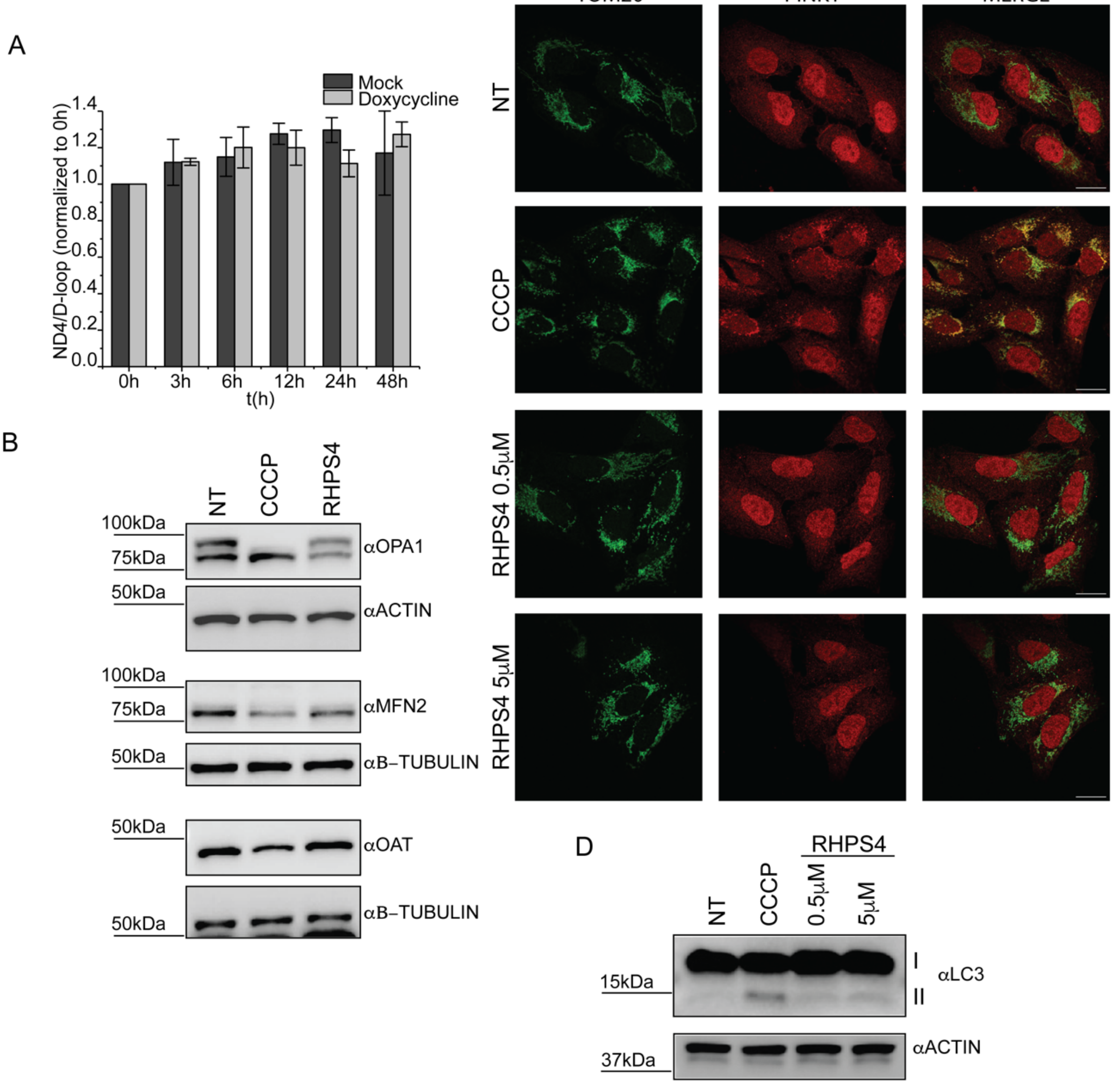

Sup. Figure 9: A. ND4/D-loop levels relative to untreated sample for HEK mitoBG4 cells treated with 0.5μM RHPS4 for the indicated time points. A constant ND4/D-loop ratio is indicative of the absence of mtDNA deletions. Data represent mean ± s.d. of three independent experiments. B. Immunoblot analysis of whole cell lysate from HEK293 cells treated with 10 μM CCCP or 0.5 μM RHPS4 for 12h. Antibodies against OPA1, MFN2 (Mitofusin 2) and OAT (Ornithine aminotransferase) were used to detect the steady state levels of the respective mitochondrial-localized proteins. C. Representative confocal images of U2OS cells treated with 20 μM CCCP or RHPS4 at the indicated concentrations and co-immunostained with the mitochondrial markers TOM20 and PINK1 (PTEN-induced kinase 1), which is recruited at the MOM upon mitophagy induction. D. Immunoblot analysis of whole cell lysate from HEK293 cells treated with 10 μM CCCP or RHPS4 at the indicated concentrations for 12h. Antibody against LC3 was used to detect the conversion of LC3 I (I) into LC3II (II), indicative of the

autophagosome formation. For B and D, B-TUBULIN and ACTIN were used as loading control. CCCP (Carbonyl cyanide m-chlorophenylhydrazone) was used as positive control of mitophagy induction.

Sup.Figure 10

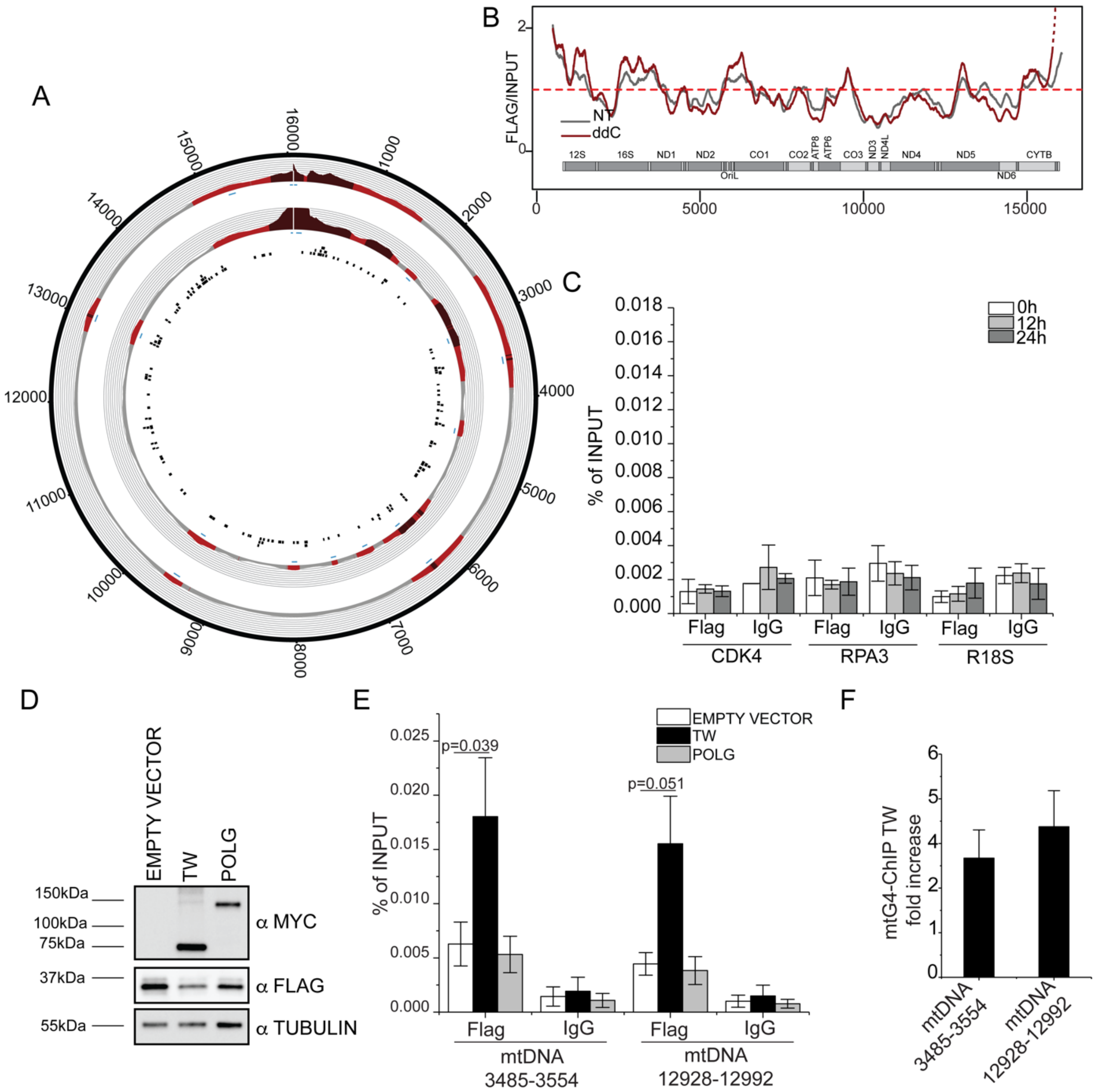

Sup.Figure 10: A. mtG4-ChIP-seq profile for induced mitoBG4 cells treated with 200 µM ddC for 3 h before pulling down with FLAG antibody. FLAG signal was normalized to the INPUT sample and expressed as ratio of FLAG vs INPUT. Areas in red indicate enrichment over INPUT samples (grey <1, light red 1 > x > 1,5 and dark red > 1,5). The blue boxes beneath the plots represent the narrow peaks that were extracted +/- 50 bp from the max ratio of the enrichment range. The black boxes in the inner circle are predicted G4 sequences (using the G4Hunter algorithm). Two biological replicates are shown. B. Linear plots showing the average FLAG to INPUT signal from the doxycycline induced replicates (in grey) and ddC treated replicates (in red). MtDNA sequence from nt 500 to nt 16 000 is displayed. C. ChIP-qPCR upon

pull down with FLAG and IgG antibodies in induced MTS-BG4 cells. Three nDNA regions were analysed. The RPA3 and CDK4 regions contain a predicted G4 sequence. Data are expressed as % of INPUT and represent mean ± s.d. of three independent experiments. D. Immunoblot analysis of INPUT samples from mtG4-ChIP in induced mitoBG4 cells over-expressing TWINKLE (TW) or POLG. Both TWINKLE and POLG contain a c-terminal myc-tag that is detected with the corresponding antibody. B-TUBULIN antibody was used as loading control. E. ChIP-qPCR upon pull down with FLAG and IgG antibodies in induced mitoBG4 cells overexpressing TWINKLE (TW) or POLG. Data are expressed as % of INPUT and represent mean ± s.d. of three independent experiments. Analysis of the data was performed using two-sample t test. F. Fold increase in mtG4-ChIP signal in TW over-expressing cells with respect to cells transfected with empty vector.

Sup.Table 1: mtG4-ChIP-seq peak enrichment for doxycycline-induced samples

Replicate 1			Replicate 2			Replicate 3			MACS 2 PEAK SUMMIT (Rep 1) Flag vs IgG	PGS	PREDICTION
Peak coordinates (Start – end)		Enrichment (Ratio Flag/Input)	Peak coordinates (Start – end)		Enrichment (Ratio Flag/Input)	Peak coordinates (Start – end)		Enrichment (Ratio Flag/Input)			
3072	3730	1,45	2491	3989	1,40	2566	3837	1,28	3528	MtDNA G4 3535	G4HUNTER (MITO 27)[a]
6106	6681	1,20	5656	6678	1,35	5839	6523	1,22	6279	MtDNA G4 6320	QGRS Mapper[b]
9281	9671	1,66	9128	9681	1,25	9409	9584	1,12	9552	MtDNA G4 9602	G4HUNTER (MITO 89)[a]
12937	13169	1,22	12870	13852	1,30	12943	13131	1,10	13034	MtDNA G4 13083	G4HUNTER (MITO123)[a]

[a] Bedrat A, Lacroix L, Mergny JL. Re-evaluation of G-quadruplex propensity with G4Hunter. *Nucleic Acids Res.* 2016;44(4):1746-1759
[b] Kikin O, D'Antonio L, Bagga PS. QGRS Mapper: a web-based server for predicting G-quadruplexes in nucleotide sequences. *Nucleic Acids Res*. 2006 Jul 1;34

Sup.Table 2: CD analysis

Oligo	Ellipticity λ_{max} (+), λ_{min} (-), nm	Topology
MtDNA G4 3535	262, 241	G4, Parallel
Scramble MtDNA G4 3535	275, 248	Non G4
MtDNA G4 6320	263, 241	G4, Parallel
Scramble MtDNA G4 6320	275,244	Non G4
MtDNA G4 9602	264, 241	G4, Parallel
Scramble MtDNA G4 9602	273, 246	Non G4
MtDNA G4 13083	264, 241	G4, Parallel
Scramble MtDNA G4 13083	272, 245	Non G4
MtDNA G4 581	295, 265	G4, antiparallel
Scramble MtDNA G4 581	293, 254	Non G4
MtDNA195	274, 248	Non G4

Sup.Table 3: Melting temperature (T_m) as function of the concentration of K^+

Oligo	Molarity K^+ (mM)	T_m (°C)
MtDNA G4 13083	150	55±0.4
	125	53±0.5
	100	50±0.5
	75	47±0.5
	50	45±0.6
	25	42±0.5
MtDNA G4 9602	150	54±1.7
	100	N/A

N/A: not available. For MtDNA G4 9602 CD melting assay at lower [K^+] was not assessed because of the high signal-to-noise ratio in the spectra at 25 ºC.

Sup.Table 4: mtG4-ChIP-seq peak enrichment for ddC and RHPS4 treated samples

ddC						RHPS4					
Replicate 1			Replicate 2			Replicate 1			Replicate 2		
Peak coordinates (Start – end)		Enrichment (Ratio Flag/Input)	Peak coordinates (Start – end)		Enrichment (Ratio Flag/Input)	Peak coordinates (Start – end)		Enrichment (Ratio Flag/Input)	Peak coordinates (Start – end)		Enrichment (Ratio Flag/Input)
2515	3896	1,53	2455	3780	1,82	2425	3789	2.46	2445	3986	2.18
5779	6568	1,54	5672	6617	1,67	5570	7168	2.01	5602	7135	1.96
9410	9657	1,22	9261	9744	1,49	9252	9837	1.58	8958	9869	1.55
12839	13256	1,52	12866	13256	1,36	12762	13307	1.37	12802	13771	1.82
						4330	4619	1.24	4350	4624	1.29
						7182	7559	1.29	7139	7534	1.38
						7883	8244	1.41	7800	8276	1.30

Sup. Table 5: Oligonucleotide used in this study

Cloning

Name	Sequence (5´-3´)
MTSBG4 For HindIII	ATATAAGCTTATGGCGTTTCTCCGAAGCATG
MTSBG4 Rev EcoRV	GAGCGATATCTTACTTGTCATCGTCATCCTTG
SOE MTS	GCACCAGCTGCACCTCTGGACAACTTGCCAAGACAGA
SOE BG4	CTTGGCAAGTTGTCCAGAGGTGCAGCTGGTGCAG

Multiplex PCR

Name	Region	Sequence (5´-3´)
D-loop For	mt 16,528 – 16,548	CTAAATAGCCCACACGTTCCC
D-loop Rev	mt 23 – 42	AGAGCTCCCGTGAGTGGTTA
D-loop Probe	mt 16,560 – 10	6FAM-CATCACGATGGATCACAGGT(NFQ)
ND4 For	mt 10,912 – 10,931	CTGTTCCCCAACCTTTTCCT
ND4 Rev	mt 10,975 – 10,994	CCATGATTGTGAGGGGTAGG
ND4 Probe	mt 10,934 – 10,951	TEX- GACCCCCTAACAACCCCC(NFQ)
B2M For	Chr15 15,798,932 – 15,798,958	GCTGGGTAGCTCTAAACAATGTATTCA
B2M Rev	Chr15 15,798,932 – 15,798,958	GCTGGGTAGCTCTAAACAATGTATTCA
B2M Probe	Chr15 15,798,969 – 15,798,984	HEX-CAGCAGCCTATTCTGC(NFQ)

qPCR

Name	Sequence (5´-3´)
G4 mtDNA 3485 F	CCCTAAAACCCGCCACATCTA
G4 mtDNA 3554 R	AGAGCGATGGTGAGAGCTAAG
G4 mtDNA 12928 F	CCACAACAAATAGCCCTTCTAAAC
G4 mtDNA 12992 R	GCTAGGAGGAGGCCTAGTAGT
mtDNA 10912 F	CTGTTCCCCAACCTTTTCCT
mtDNA 10994 R	CCATGATTGTGAGGGGTAGG
mtDNA 8581	GCCGCAGTACTGATCATTC
mtDNA 8729	CAGGTTCGTCCTTTAGTGTTG

r18S F (nDNA)	ACGGACCAGAGCGAAAGCAT
r18S R (nDNA)	GGACATCTAAGGGCATCACAGAC
RPA3 FOR (nDNA)	CGGAAGTTGACAGATACAGGG
RPA3 REV (nDNA)	GATCGCAGAAAGGTAGTCTCAG
GAPDH FOR (nDNA)	GCTACTAGCGGTTTTACGGGCG
GAPDH REV (nDNA)	TGCGGCTGACTGTCGAACAGG
CDK4 FOR (nDNA)	CCACCCTCACCATGTGACC
CDK4 REV (nDNA)	CTTACACTCTTCGCCCTCCTC
**Taq Polymerase STOP assay**	
Primer 5´-3´	TET-TGAAAACATTATTAATGGCGTCGAGCGTCCG
mtDNA581 G4 PolG ext	TAAACTGTGGGGGGTGTCTTTGGGGTTTGGTTGGTTCGGGGTATGGGGTTAGCAGCGGTATATCGGACGCTCGACGCCATTAATAATGTTTTCA
mtDNA195 NonG4 PolG ext	TTACTAAAGTGTGTTAATTAATTAATGCTTGTAGGACATAATAATAACAATATATCGGACGCTCGACGCCATTAATAATGTTTTCA
**CD and ELISA**	
Name	Sequence (5´-3´)
MtDNA G4 3535	AGGTCGGGGCGGTGATGTAGAGGGTGATGGT
Scramble MtDNA G4 3535	AGtGCGGtGaGCGGTGAGTGAGaGGTGTGGT
MtDNA G4 6320	GGGTGGGAGTAGTTCCCTGCTAAGGGAGGG
Scramble MtDNA G4 6320	AGtGtGTGtGccGAGAGTGCTGaGaGAGcGtG
MtDNA G4 9602	TAGGAGTGGGACTTCTAGGGGATTTAGCGGGGTGATGCCTGTTGGGGGC
Scramble MtDNA G4 9602	TGaGAGTGcGtGATGcGatGttGAGCGtGaGtGTGAGCGTGcGtGGtcG
MtDNA G4 13083	TGAGTGGAGTAGGGCTGAGACTGGGGTGGGGCCTTCTATGGCTGAGGGG
Scramble MtDNA G4 13083	GAGTGtGAGAGaGcGTGAGTGcGaGtGTGcGGcGTTGtGCGAGtGcGtG
MtDNA G4 581	GGGGTGTCTTTGGGGTTTGGTTGGTTCGGGGTATGGGG
Scramble MtDNA G4 581	GCGTGTGTGTGTGTGTGTGTGTGTGTGTGCGGAGTGGTGG
MtDNA 195 (non G4)	TTACTAAAGTGTGTTAATTAATTAATGCTTGTAGGACATAATAATAACAA (modified with Biotin TAG at 5´-end for ELISA)
HIF1 G4	BioTeg-GCGCGGGGAGGGGAGAGGGGGCGGGAGCGCG

Sup. Table 6: Antibodies used in this study

Ab (vs)	*Company*			*Cat. num*	
DNA G4 (clone bg4)	Customized			---------	
Immunoblot (Clean Blot and True Blot antibody used for detection of IP samples)					
Ab (vs)	*Company*	*Cat. num*	*dilution*	*II Antibody*	*dilution*
Flag M2	Sigma-Aldrich	F1804	1:1000	Goat anti-Mouse IgG (H+L) HRP (Thermo Scientific)	1:10000
PolGA	Abcam	Ab128899	1:1000	Clean Blot IP HRP (Thermo Scientific)	1:1000
B-Tubulin	Rabbit	#2128	1:1000	Goat anti-Rabbit IgG (H+L) HRP (Thermo Scientific)	1:10000
GAPDH	Cell Signalling	#2118	1:1000	Goat anti-Rabbit IgG (H+L) HRP (Thermo Scientific)	1:5000
LAMIN A/C	Cell Signalling	#4777	1:1000	Goat anti-Mouse IgG (H+L) HRP (Thermo Scientific)	1:3000
MFN2	Protein Tech	12186-1-AP	1:5000	Goat anti-Rabbit IgG (H+L) HRP (Thermo Scientific)	1:20000
LC3	MBL	PM036	1:5000	Goat anti-Rabbit IgG (H+L) HRP (Thermo Scientific)	1:20000
OAT	Origene	AMO9362PU	1:5000	Goat anti-Mouse IgG (H+L) HRP (Thermo Scientific)	1:20000
PrimPol	Customized	---------	1:1000	Clean Blot IP HRP (Thermo Scientific)	1:1000
B-Actin	Sigma	A5441	1:10000	Mouse TrueBlot ULTRA HRP (Rockland)	1:2000
C23 MS-3	Santa Cruz	Sc-8031	1:1000	Mouse TrueBlot ULTRA HRP (Rockland)	1:2000
Twinkle	Abcam	ab187517	1:1000	Clean Blot IP HRP (Thermo Scientific)	1:1000
SSBP1	ProteinTech	12212-1-AP	1:1000	Clean Blot IP HRP (Thermo Scientific)	1:1000
				Goat anti-Rabbit IgG (H+L) HRP (Thermo Scientific)	1:3000
TOM20	SantaCruz	Sc-11415	1:1000	Clean Blot IP HRP (Thermo Scientific)	1:1000
TOM20 (F10)	SantaCruz	Sc-17764	1:1000	Goat anti-Mouse IgG (H+L) HRP (Thermo Scientific)	1:3000
OPA1	BD Bioscience	612606	1:1000	Mouse TrueBlot ULTRA HRP (Rockland)	1:2000
				Goat anti-Rabbit IgG (H+L) HRP (Thermo Scientific)	1:3000
TFAM	ProteinTech	22586-1-AP	1:1000	Clean Blot IP HRP (Thermo Scientific)	1:1000
PCNA	Cell Signalling	#2586	1:1000	Mouse TrueBlot ULTRA HRP (Rockland)	1:2000
ICC					
Ab (vs)	Company	Cat. num	dilution	II Antibody	dilution
Flag M2	Sigma-Aldrich	F1804	1:500	Goat anti Mouse IgG (H+L) –Alexa Fluor594 (Molecular Probes)	1:1000

TOM20	SantaCruz	Sc-11415	1:200	Goat anti Rabbit IgG (H+L) - Alexa Fluor488 (Molecular Probes)	1:500
TOM20	SantaCruz	Sc-17764	1:100	Goat anti Mouse IgG (H+L) –Alexa Fluor594 (Molecular Probes)	1:100
TFAM	ProteinTech	22586-1-AP	1:100	Goat anti Rabbit IgG (H+L) - Alexa Fluor488 (Molecular Probes)	1:500
SSBP1	ProteinTech	12212-1-AP	1:100	Goat anti Rabbit IgG (H+L) - Alexa Fluor488 (Molecular Probes)	1:500
PINK1	Novus biologicals	BC100-494	1:100	Goat anti Rabbit IgG (H+L) - Alexa Fluor594 (Molecular Probes)	1:100

IP				
Antibody	Company	Cat. num	Amount	Host
Flag M2	Sigma-Aldrich	F1804	10ug	Mouse
Mouse IgG	Santa Cruz	Sc-2025	10ug	Mouse
PolGA	Invitrogen	PA521314	5ug	Rabbit
TFAM	Proteintech	22586-1-AP	1ug	Rabbit
Twinkle	Abcam	Ab187517	5ug	Rabbit
mtSSB	Proteintech	12212-1-AP	1ug	Rabbit
PrimPol Neutron	Customized	--------------	5ug	Rabbit
Tom20	Santa Cruz	SC-11415	1ug	Rabbit
Rabbit IgG	Santa Cruz	Sc-2027	5ug	Rabbit
